# Physical space interacts with clonal fragmentation and nutrient availability to affect the growth of *Salvinia natans*

**DOI:** 10.1371/journal.pone.0226850

**Published:** 2019-12-23

**Authors:** Chao Si, Yu Jin, Jing Lin, Jian-Feng Zhang, Jin-Song Chen, Fei-Hai Yu

**Affiliations:** 1 School of Nature Conservation, Beijing Forestry University, Beijing, China; 2 Institute of Wetland Ecology & Clone Ecology, Taizhou University, Taizhou, China; 3 College of Life Science, Sichuan Normal University, Chengdu, China; Shandong University, CHINA

## Abstract

Physical space, clonal fragmentation and nutrient availability can each affect the growth of clonal plants, but their interactive effect has been little studied. We grew un-fragmented (connected) and fragmented (disconnected) ramet pairs of the floating, clonal plant *Salvinia natans* in cylindrical containers with different diameters and heights (volumes) filled with solutions of two nutrient levels (high vs. low). To simulate competition environments that are commonly confronted by *S*. *natans*, we also added two ramets of another floating plants *Spirodela polyrrhiza* in each container. Biomass (total biomass, floating biomass and submerged biomass) and number of ramets of *S*. *salvinia* were higher in the containers with a larger diameter. Compared to the low nutrient level, the high nutrient level increased number of ramets, and altered submerged to floating mass ratio of *S*. *salvinia*. The impacts of physical space on floating mass and number of ramets were stronger under the high than under the low nutrient level. Clonal fragmentation positively affected biomass in the containers with a smaller volume (a smaller height and diameter), but had little impact in the containers with a larger volume (a larger height or diameter). Our results suggest that physical space can interact with nutrients and clonal fragmentation to affect the performance of *S*. *salvinia* under competition.

## Introduction

Aquatic ecosystems are commonly dominated by clonal plants [1–4]. Many clonal plants can form a network of ramets interconnected by stolons, rhizomes or horizontally growing roots [5–8]. Aquatic ecosystems are frequently subjected to disturbances caused by natural events and human activities such as flooding, invertebrate and waterfowl grazing, fishing and transportation [3,9–11], which can fragment the previous interconnected clonal network of aquatic plants [10,12,13]. Fragmentation may adversely affect plant growth because transport of resources such as carbohydrates and mineral nutrients between ramets is cut off [14–16]. However, fragmentation may increase ramet production by releasing apical dominance and increasing branching frequency [4,17]. Assessing responses of aquatic plants to disturbance-mediated clonal fragmentation can deepen our understanding of plant strategies in aquatic habitats, and provide scientific support for the restoration of degraded aquatic plant communities [3,6].

Physical space is considered a resource that may affect the growth, morphology, physiology and reproduction of plants [18–20]. Plants cultured in larger soil volume or depth are generally taller, have thicker stems and wider canopies, form more branches, and produce more and larger leaves [21]. However, for floating plants the effect of physical space on their growth may be predominantly determined by the area of the water surface as it may greatly affect crowding and thus the availability of light, nutrients and O_2_ [10,22]. If floating plants are sensitive to nutrient availability, then the volume of physical space may also play a role as a larger volume of water may contain more nutrients.

Nutrient availability can also greatly influence the growth of floating plants [23]. For instance, increasing nutrient availability can increase the growth of water hyacinth (*Eichhornia crassipes*) [24], water cabbage (*Pistia stratiotes*) [25] and duckweed (*Lemna major*) [26]. In addition, nutrient availability may interact with clonal fragmentation to affect the growth of aquatic plants, and the negative impact of clonal fragmentation may be less important when nutrient availability is higher. Meanwhile, nutrient availability may also interact with physical space to influence the growth of floating plants as higher nutrient availability may accelerate the growth of floating plants and thus increase their crowing when the water surface is limited.

Also, physical space may interact with clonal fragmentation to affect the growth of floating plants. This hypothesis is because fragmentation may increase branching frequency and ramet production [4,17]. Increasing branching and ramet density may greatly increase crowding and thus intensify intraspecific competition when the surface area of water is limited so that individual ramet growth of the floating plant can greatly decrease. Conversely, when the surface area of water is unlimited, then fragmentation may greatly increase its ramet growth and spread.

To test these hypotheses, we grew fragmented (disconnected) or un-fragmented (connected) ramet pairs of a stoloniferous clonal fern *Salvinia natans* in cylindrical containers differing in diameter and depth (thus differing in water surface area and volume) and filled with solutions of two different nutrient concentrations. As *S*. *natans* commonly co-occurs with another floating plant *Spirodela polyrrhiza*, we also added the same number of ramets of *S*. *polyrrhiza* to each container to simulate competition environments that are commonly faced by this species. Specifically, we addressed the following questions. (1) Does clonal fragmentation affect the growth of *S*. *natans*? We predict that clonal fragmentation will negatively affect the growth of *S*. *natans* as it prevents resource translocation and sharing between ramets. (2) Does physical space affect the growth of *S*. *natans*? We expect that increasing the diameter and depth of the containers will increase the growth of *S*. *natans*. (3) Does nutrient availability interact with physical space or clonal fragmentation to impact the growth of *S*. *natans*? We expect that the negative impact of clonal fragmentation will be less under high than under low nutrient availability and that the impact of physical space will be more severe under high than under low nutrient availability. (4) Does physical space interact with clonal fragmentation to affect the growth of *S*. *natans*? We expect that the negative effect of fragmentation will be aggravated when physical space is limited.

## Materials and methods

### Plant species

*Salvinia natans* (L.) All. is an aquatic free-floating, clonal fern [27]. It can reproduce rapidly on water surface [27] and is widely distributed in Asia, Europe, and introduced in North America [28,29]. This species produces stolons along which interconnected ramets are produced. Each ramet of *S*. *natans* consists of two green floating fronds about 0.8–1.4 cm long and 0.5–0.8 cm wide, and a brown, heavily dissected, rootlike submerged frond which probably functions as roots and also acts as a stabilizer [28].

*Spirodela polyrrhiza* (L.) Schleid is also a free-floating, clonal plant [30]. It is widely distributed in temperate and tropical regions of the world [30]. The leaves of *S*. *polyrhiza* are obovate, 5–8 mm long and 4–6 mm wide, whose upper side (contacting air) is green and downward side (contacting water) is purple. From the central part of a leaf on the downward site, 5–11 roots are formed with 3–5 cm in length. This species is commonly found to grow together with *S*. *natans* and *Lemna minor*, forming floating plant communities in freshwater ecosystems in the south of China [30,31].

### Plant material collection and preparation

Plants of *S*. *natans* and *S*. *polyrrhiza* were collected from a small pond (28°39′N, 121°23′E) in Jiaojiang District, Taizhou, Zhejiang province, China. The sampling site did not belong to any farms or national parks and also did not involve any endangered or protected species, so we did not need any relevant permission for collecting plant samples. The plants were brought to a greenhouse at Taizhou University for cultivation for several days before the experiment. For *S*. *natans*, ramet pairs consisting of the 4^th^ and 5^th^ ramets to the stolon apex, which were interconnected by a stolon internode, were cut off from the rest of the clones and used for target plants. The fronds of these ramets were already fully developed. For *S*. *polyrrhiza*, similar sized ramets were selected and used as competitor plants.

### Experimental design

To quantify space, we used three types of cylindrical containers, i.e. 8 cm in diameter × 12 cm in depth (coded as S), 16 cm diameter × 12 cm in depth (W) and 8 cm in diameter × 42 cm in depth (T). The level of nutrient solution for cultivation in each container was maintained 2 cm below the upper edge of the container so that the volume of nutrient solution in the containers of W and T was the same, which was four times that in the containers of S. Containers of each type were each filled with either 1% or 10% of full strength Hoagland solution (N, P concentration of full-strength Hoagland’s solution were 210 mg L^−1^ and 31 mg L^−1^) [32] grown with a pair of ramets of *S*. *natans* whose stolon connection was either severed in the midway (fragmented) or kept intact (not fragmented), making 12 treatments. Two ramets of *S*. *polyrrhiza* were placed in each container as the competitor plants. Each treatment was replicated eight times, making 96 containers in total.

The experiment was conducted in the same greenhouse for material cultivation. It started on 28 April 2018, and ended on 29 May 2018, lasting 32 days. During the experiment, the mean air temperature in the greenhouse was 24 °C, and the mean air humidity was 82%. Distilled water was added to compensate for the loss due to evaporation and absorption. To avoid the connection breakage of *S*. *natans* caused by disturbance, we did not refill the nutrient solutions, but carefully removed the algae every day.

### Measurements and data analysis

At the end of the experiment, we counted ramets of *S*. *natans* in each container. The plants were separated into floating fronds and submerged parts (submerged fronds plus stems), then dried at 70 °C for 72 h and weighed. We calculated the ratio of submerged mass to floating mass.

We used three-way ANOVA to test the effects of physical space (S, W and T), nutrient level (1 and 10% Hoagland solution), clonal fragmentation (with and without) and their interactions on total mass, floating mass, submerged mass, the ratio of submerged mass to floating mass, and number of ramets of *S*. *natans*. Plants in two containers with the high nutrient level and no fragmentation were lost: one was in the container type of S and one in the container type of W. All analyses were conducted using SPSS 22.0 (Chicago, IL, USA).

## Results

**Fig 1:**
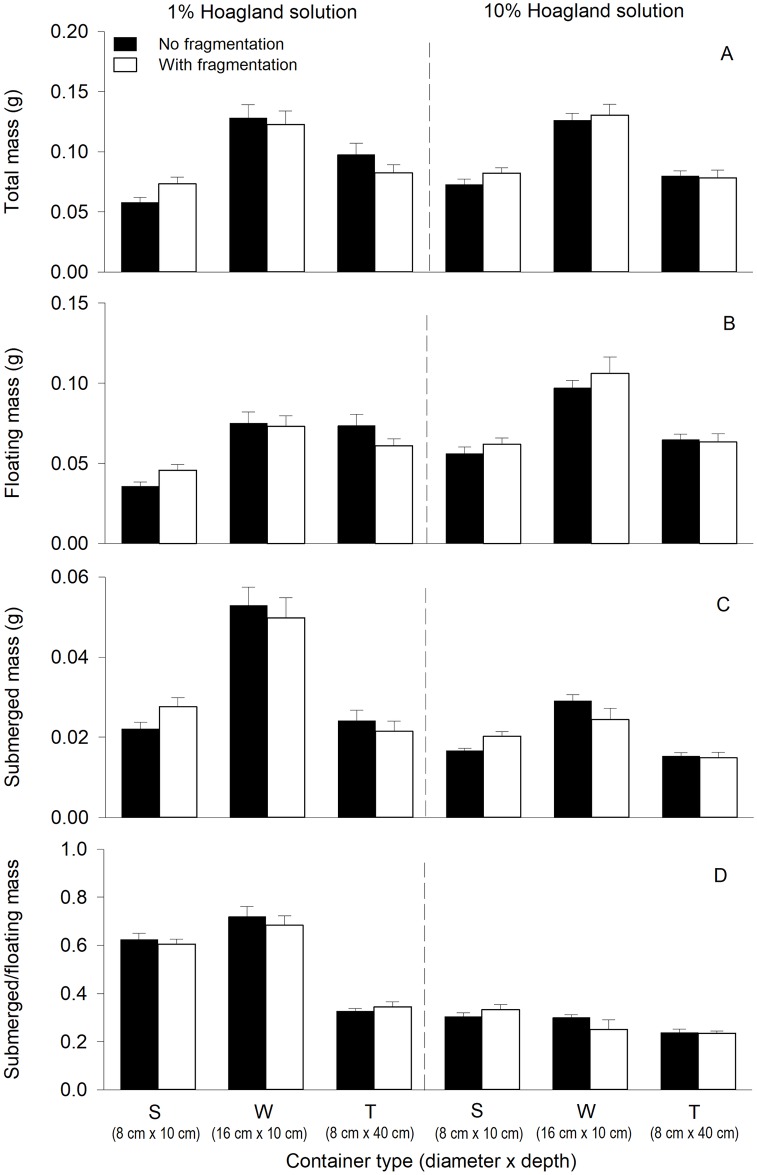
Effects of physical space (container type), nutrient level and clonal fragmentation on total mass (A), floating mass (B), submerged mass (C), and submerged to floating mass (D) of *Salvinia natans*. Bars and vertical lines are means and SE (n = 8).

Physical space significantly affected all measures of *S*. *natans* (main effect of physical space in Table 1). In general, biomass (total mass, floating mass and submerged mass) and ramet number *S*. *natans* were greater in W than in S and T (Fig 1A–1C). The ratio of submerged mass to floating mass were higher in S and W than in T (Fig 1D).

**Table 1:** Effects of physical space (container type), nutrient level and clonal fragmentation (Fragment.) on the growth of *Salvinia natans*.

Effect	d.f.	Total mass	Floating mass	Submerged mass	Submerged/floating mass	No. of ramets
Space (S)	2	59.83***	49.79***	65.09***	45.59***	39.89***
Nutrient (N)	2	0.63^ns^	23.62***	72.00***	237.10***	54.22***
Fragment. (F)	1	0.31^ns^	0.47^ns^	0.03^ns^	0.86^ns^	0.74^ns^
S × N	2	3.69*	8.18**	4.41*	17.79***	1.13^ns^
S × F	2	3.41*	*2*.*99*^*#*^	4.16*	2.10^ns^	1.45^ns^
N × F	1	0.09^ns^	0.08^ns^	0.08^ns^	0.41^ns^	0.23^ns^
S × N × F	2	0.60^ns^	0.84^ns^	0.37^ns^	1.27^ns^	0.38^ns^
Error	110					

The given are degree of freedom (d.f.), F values and the significance levels (*** *P* < 0.001; ** *P* < 0.01; * *P* < 0.05; ^#^ *P* < 0.1; ^ns^ > 0.1) of ANOVA.

**Fig 2:**
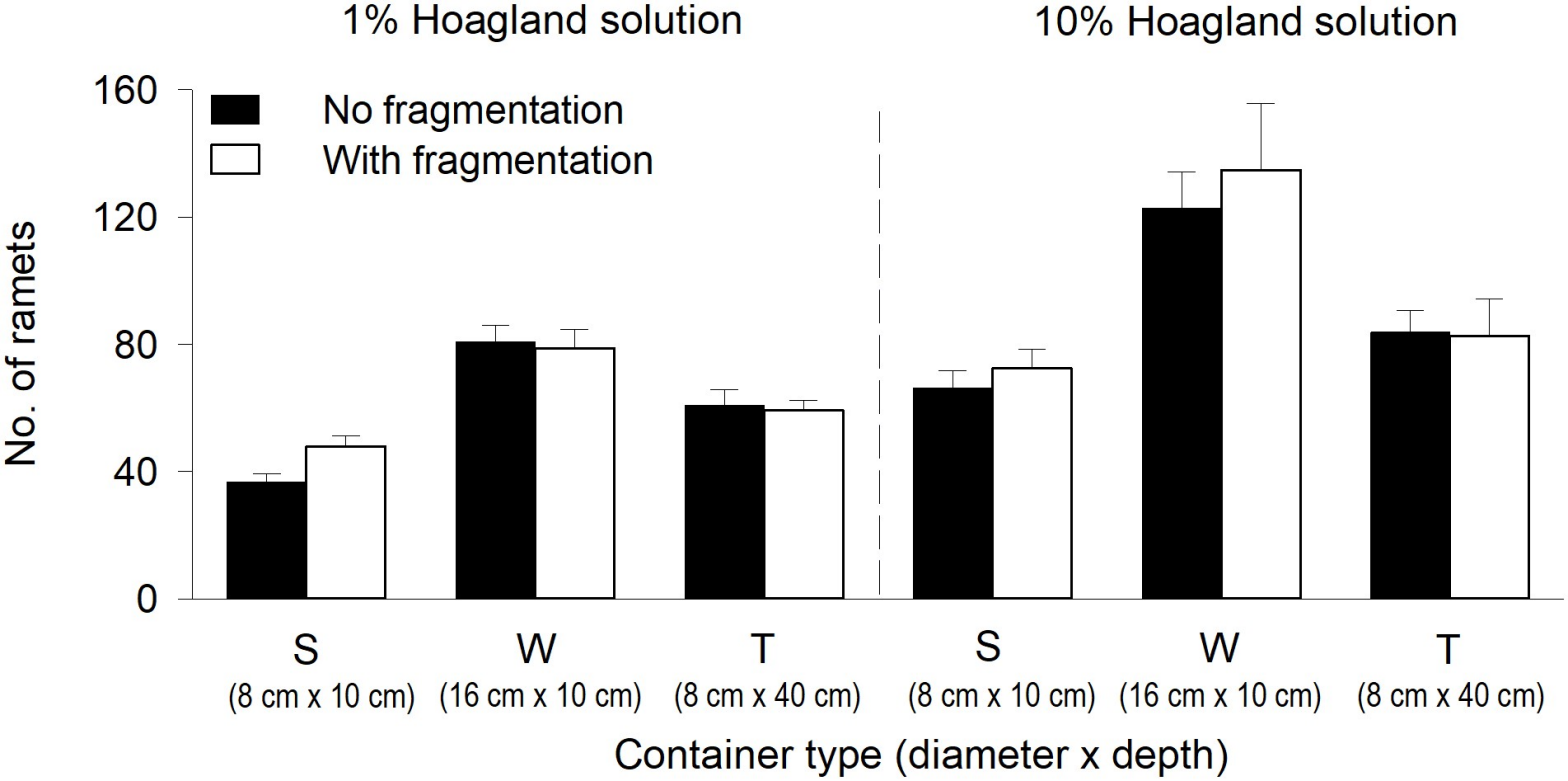
Effects of physical space (container type), nutrient level and clonal fragmentation on number of ramets of *Salvinia natans*. Bars and vertical lines are means and SE (n = 8).

Compared to the low nutrient level (1% Hoagland solution), the high nutrient level (10% Hoagland solution) tended to increased total mass in S, decreased that in T and did not change it in W, as indicated by the significant interaction of physical space × nutrient level (Table 1, Fig 1A). Compared to the low nutrient level, the high nutrient level increased floating mass in both S and W, and tended to decreased that in T (significant physical space × nutrient level effect and significant nutrient effect in Table 1, Fig 1B). By contrast, the high nutrient level decreased submerged mass in all three levels of physical space, but such an impact was higher in W than in S and T (significant physical space × nutrient level effect and significant nutrient effect in Table 1, Fig 1B). The high nutrient level decreased the ratio of submerged mass to floating mass, but such an impact was much higher in S and W than in T (significant physical space × nutrient level effect and significant nutrient effect in Table 1, Fig 1B). The high nutrient level increased number of ramets (Fig 2).

Clonal fragmentation increased total mass, floating mass and submerged mass only in S, but had not impact on biomass in W or T (significant or marginally significant physical space × clonal fragmentation effect in Table 1, Fig 1A–1C). Clonal fragmentation did not significantly affect the ratio of submerged to floating mass or number of ramets (Table 1, Figs 1D and 2).

## Discussion

Physical space significantly affected the growth and biomass allocation of the clonal floating plant *S*. *natans*. This result is consistent with previous findings and suggests that physical space can influence the performance of plants [18–20,22]. In the container with a larger diameter and thus a large surface area of water, *S*. *natans* produced more biomass and ramet number (Fig 1). The likely reason is that the larger surface of water could provide clonal floating plants with more space to reproduce vegetatively [33,34]. In addition, the larger surface could decrease the intensity of competition resulting from crowding, which could indirectly promote plant growth and clonal reproduction [35–37].

As expected, nutrient levels affected the growth and biomass allocation of *S*. *natans*. At the higher nutrient level, *S*. *natans* tended to have higher floating mass, but lower submerged mass, thereby resulting in the smaller ratio of submerged mass to floating mass. The change in biomass allocation pattern is consistent with the optimal theory of biomass allocation [38–40], i.e. increasing biomass allocation to the plant part with the most limiting resources and decreasing that to the plant part with abundant resources [41–44]. In addition, the high nutrients level increased number of ramets, indicating that the high nutrient level benefits clonal reproduction, agreeing with findings of many previous studies [16,27,45,46]. However, total mass under the high nutrient level was not significantly different from that under the low nutrient level. One possible reason is that the high nutrient level greatly increased the abundance and thus competitive intensity of the competitor (*S*. *polyrrhiza*), which reduced the size of the ramets of *S*. *natans*. As a result, the high nutrient level did not increase total mass of *S*. *natans*.

Clonal fragmentation is generally considered an important factor that can regulate the growth and spread of aquatic clonal plants [3,10,47]. Many studies have shown that clonal fragmentation could decrease the growth of clonal plants because it hindered resource translocation between ramets [7,48–50] or have no significant effect because the cost to the ramets exporting resources counteracted the benefits to the ramets importing resources [17,51,52]. Unexpectedly, we found that clonal fragmentation increased the growth of *S*. *natans* when it was grown in the containers with a small volume (in S with a small diameter and depth). One likely reason is that clonal fragmentation released apical dominance, which increased branching intensity and offspring ramet production and thus enhanced the growth of the whole plant [6,10,53]. Another possible explanation is that the fragmentation-mediated growth increase in the donor ramet (exporting resources) was more than the fragmentation-mediated growth decrease in the recipient ramet (importing resources) [53–55]. Also, there may be great maintenance costs associated with connective tissues between ramets, which may contribute to the decreased growth of the plant without clonal fragmentation [56]. However, clonal fragmentation had no impact on *S*. *natans* when it was grown in the containers with a larger volume (in W with a larger diameter or in T with a large depth). These results suggest that the impact of clonal fragmentation on the growth of the floating plant *S*. *natans* varied with physical space where the plant could occupy. However, we still do not know the mechanism underlying such a space-dependent effect of clonal fragmentation, and further studies could be designed to test the potential mechanisms.

We found that physical space interacted with nutrient availability to affect the growth of *S*. *natans*. For instance, the difference in floating mass of *S*. *natans* between the treatment in which it was grown in the containers with a larger diameter but a smaller depth (W) and the treatment in which it was grown in the containers with a smaller diameter but a larger depth (T) was much greater when the nutrient level was high than when it was low (Fig 1B), and the difference in submerged mass showed the opposite pattern (Fig 1C). Also, the ratio of submerged to floating mass differed more between the container types when the nutrient level was low than when it was high (Fig 1D). These results suggest that the impact of physical space on plant performance could vary with nutrient availability [20]. We do not know the exact mechanism underlying the interaction effect of physical space × nutrient availability. One possible explanation is that the response of the submerged fronds (functioning as roots) of *S*. *natans* to nutrient availability could be restricted by physical space. For instance, increasing the nutrient level decreased the number and length of submerged fronds of *S*. *natans* much more when *S*. *natans* was grown in the containers with a larger diameter but a smaller depth (W) than when it was grown in the containers with a smaller diameter but a larger depth (T) (Chao Si personal observation). High nutrient availability commonly increases branching and ramet production [57–59], as also observed in our study (Fig 2). When physical space is limited, increasing ramet number is expected to increase crowding and thus intensify intra- and/or interspecific competition to reduce plant performance [10,60–62]. By contrast, if physical space is unlimited, increasing ramet number may have little or a positive effect on plant performance [63]. Therefore, physical space may interact with nutrient availability to affect plant performance [20]. However, in our study the interaction effect of physical space and nutrient availability on *S*. *natans* cannot be explained by this mechanism as floating and submerged mass showed opposite patterns (as described in the preceding paragraph). The underlying mechanism is unclear and deserves further studies.

We conclude that physical space can interact with clonal fragmentation and nutrient availability to affect the performance of clonal floating plants. However, we still lack a mechanistic understanding of such a context-dependent effect of physical space. Studies that are designed to examine such underlying mechanisms are promising to give insights into the contribution of physical space on plant performance.
